# Validation of Web-Based Physical Activity Measurement Systems Using Doubly Labeled Water

**DOI:** 10.2196/jmir.2253

**Published:** 2012-09-25

**Authors:** Hideyuki Namba, Yukio Yamaguchi, Yosuke Yamada, Satoru Tokushima, Yoichi Hatamoto, Hiroyuki Sagayama, Misaka Kimura, Yasuki Higaki, Hiroaki Tanaka

**Affiliations:** ^1^Faculty of Sports and Health ScienceFukuoka UniversityFukuokaJapan; ^2^Kyoto Prefectural University of MedicineKyotoJapan; ^3^Japan Society for the Promotion of ScienceTokyoJapan

**Keywords:** Physical activity, energy expenditure, doubly labeled water, Japan

## Abstract

**Background:**

Online or Web-based measurement systems have been proposed as convenient methods for collecting physical activity data. We developed two Web-based physical activity systems—the 24-hour Physical Activity Record Web (24hPAR WEB) and 7 days Recall Web (7daysRecall WEB).

**Objective:**

To examine the validity of two Web-based physical activity measurement systems using the doubly labeled water (DLW) method.

**Methods:**

We assessed the validity of the 24hPAR WEB and 7daysRecall WEB in 20 individuals, aged 25 to 61 years. The order of email distribution and subsequent completion of the two Web-based measurements systems was randomized. Each measurement tool was used for a week. The participants’ activity energy expenditure (AEE) and total energy expenditure (TEE) were assessed over each week using the DLW method and compared with the respective energy expenditures estimated using the Web-based systems.

**Results:**

The mean AEE was 3.90 (SD 1.43) MJ estimated using the 24hPAR WEB and 3.67 (SD 1.48) MJ measured by the DLW method. The Pearson correlation for AEE between the two methods was *r *= .679 (*P *< .001). The Bland-Altman 95% limits of agreement ranged from –2.10 to 2.57 MJ between the two methods. The Pearson correlation for TEE between the two methods was *r *= .874 (*P *< .001). The mean AEE was 4.29 (SD 1.94) MJ using the 7daysRecall WEB and 3.80 (SD 1.36) MJ by the DLW method. The Pearson correlation for AEE between the two methods was *r *= .144 (*P *= .54). The Bland-Altman 95% limits of agreement ranged from –3.83 to 4.81 MJ between the two methods. The Pearson correlation for TEE between the two methods was *r *= .590 (*P *= .006). The average input times using terminal devices were 8 minutes and 10 seconds for the 24hPAR WEB and 6 minutes and 38 seconds for the 7daysRecall WEB.

**Conclusions:**

Both Web-based systems were found to be effective methods for collecting physical activity data and are appropriate for use in epidemiological studies. Because the measurement accuracy of the 24hPAR WEB was moderate to high, it could be suitable for evaluating the effect of interventions on individuals as well as for examining physical activity behavior.

## Introduction

Despite the known hazards of physical inactivity, it continues to be a major risk in the development of chronic diseases [1]. Physical inactivity is associated with increased incidence rates of obesity, diabetes, cardiovascular diseases, osteoporosis, and cancer [2-4]. Therefore, it is important that instruments be developed that allow accurate measurement of the level of physical activity in the population.

Physical activity questionnaires are the most frequently used instruments in epidemiological studies for estimating physical activity or energy expenditure [5]. However, the validity of traditional self-reported questionnaires is low when compared with the ideal doubly labeled water (DLW) method [6]. In addition, the tasks associated with conventional questionnaires, including collection and analysis, require time and effort. The validity of the triaxial accelerometer is higher than that of questionnaires when compared with the DLW method [7]. However, it is difficult to measure cycling, swimming, and activities involving only the upper limb using an accelerometer [5]. Accelerometers are easily available and include memory for long-term data collection. However, because they are expensive, they cannot be used for epidemiological studies that require physical activity or energy expenditure measurements in large populations. The DLW method is one of the most accurate and valid systems used for evaluating total energy expenditure (TEE) under free-living conditions [8]. The method can also be used to assess physical activity energy expenditure (AEE) and physical activity level (PAL) in association with measurement of basal metabolic rate (BMR) [9,10]. However, because it too is expensive, the DLW method is not suitable for epidemiological studies.

Therefore, it is necessary to develop new, inexpensive methods of evaluating the levels of physical activity in large populations. Web-based measurement systems have been suggested as being convenient for collecting self-reported physical activity data compared with traditional questionnaires. In addition, Web-based systems may improve measurement accuracy because of the interactive communication associated with responses to questions. Responses can be obtained from individuals anywhere and at any time because Web-based systems can be completed using terminal devices, such as cell phones and smartphones. Two recent studies validated Web-based systems for measuring physical activity against the DLW method and found their validity was equivalent to that of traditional questionnaires [11,12]. More accurate Web-based systems for measuring physical activity are required. The successful and systematic collection of demographic and lifestyle data is central to any epidemiological study. Therefore, such technologies as the Internet and mobile phones have great potential for use in this kind of study [13]. The purpose of the present study was to develop two Web-based physical activity systems and examine their validity against the DLW method.

## Methods

### Participant Recruitment and Exclusion Criteria

We recruited 20 healthy people (25–61 years, 10 men and 10 women) to participate in the study. Participants were recruited by email through advertisements in a local email newsletters of 5000 registrants and a campus of the university in Fukuoka, Japan.

The participants received a ¥10,000 gift certificate for participating in the experiments. Inclusion criteria were that each participant should have one of the following: smartphone, tablet device, personal computer, or mobile phone; they also had to have Internet and email access. Exclusion criteria were metabolic disease, pregnancy, currently lactating, or being a competitive athlete; this was because these conditions alter normal energy expenditure. The study was conducted with the approval of the Ethics Committee of Fukuoka University, Fukuoka, Japan. All participants received a full explanation of the study’s purpose and content, and we confirmed that they understood this before they gave their written consent.

### Study Overview

We developed two Web systems that functioned via a website and allowed for the automatic delivery of email. Diary [14] and 7-day recall [15] methods, developed previously, were adjusted so that they could be completed online. We named these the 24-hour Physical Activity Record Web version (24hPAR WEB) and the 7 days Recall Web version (7daysRecall WEB), respectively. The study took place over 2 weeks, and participants were requested to respond to an email sent at 8:00 every evening before bedtime using the terminal device of their choice.

**Figure 1:**
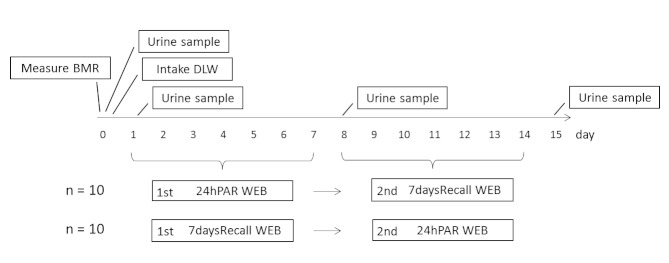
Schedule for measurement of doubly labeled water (DLW) and delivery of the Web-based systems over a 2-week period. 7daysRecall WEB = 7 days Recall Web, 24hPAR WEB = 24-hour Physical Activity Record Web, BMR = basal metabolic rate.

Figure 1 shows the time schedule for the DLW and Web-based system measurements. For the first week, 10 participants were randomly selected to complete the 24hPAR WEB, with the remaining 10 participants answering the 7daysRecall WEB. In the second week, the participants used the Web-based system that they had not completed during the first week. To determine the validity of the systems, we calculated TEE, AEE, and PAL each week. TEE was determined using the DLW method, while AEE and PAL were based on the calculation of BMR from expired gas analysis.

### Web-Based Physical Activity Measurements

The diary method [14] required that participants record activities performed in each of four different categories (work-related activities, way to work, leisure-time activities, and sports activities) every 15 minutes for a cross-tabulation. In total, there was a choice of 66 different activity behaviors among the four categories. Multimedia Appendix 1 shows a sample screen of the 24hPAR WEB activities and a list of their metabolic equivalents (METs). For every 15 minutes over each 24-hour period for 1 week, participants chose the behavior on the left of the screen and then completed the table on the right. For the 24hPAR WEB, we added an extra 33 types of behavior compared with the original 33 [14] to ensure that we included a greater variety of lifestyle behaviors. We included behaviors suggested by the NHK’s (Japan Broadcasting Corporation) National Lifetime Study 2010 [16] and the Sasakawa Sports Foundation’s 2010 National Sports-Life Survey [17] as being common to Japanese adults. We determined the intensity (in METs) of the new activities based on previous research [18]. Response results, expressed as the intensity of each activity, were recorded on a Web server every 15 minutes.

We explained to the participants how to use the system during a briefing session. Since this system operates in JAVA script, launching the software requires access to the URL of the email address that was registered.

The TEE (in MJ) for each 24-hour period was calculated using the following equation: BMR × 24-hour average METs × 1.1 ÷ 0.9 × 4.184 × 10^–3 ^[19].

The AEE (in MJ) for each 24-hour period was calculated using the following equation: TEE × 0.9 – BMR.

BMR was the value obtained from expired gas analysis. The constant 1.1 reflected the resting metabolic rate for sitting and was equivalent to an increase of approximately 10% of the BMR. The resting metabolic rate in the sitting position (1 MET) is different from the supine position BMR with fasting. The constant 0.9 reflected dietary thermogenesis of approximately 10%, and the conversion factor of 4.184 × 10^–3 ^was used to transform the values from kilocalories to megajoules.

The 7-day recall method [15] requires an individual to recall the previous 7 days’ physical activity behavior during an interview. In contrast, the participants in the present study had to input data into the 7daysRecall WEB system every day for 1 week. The day was divided into three periods—morning, afternoon, and evening—and the participants had to recall their physical activity during those periods as well as the intensity of that activity. Multimedia Appendix 2 describes the 7daysRecall WEB, the input screen, illustrations of the activities, and their intensity. The activity intensity was divided as follows: light (1.5-2.9 METs), moderate (3.0-3.9 METs), moderate to high (4.0-5.9 METs), high (6.0-7.9 METs), and very high (≥8.0 METs). The participants were able to select the intensity of the physical activity based on illustrations that demonstrated the differences between the various intensity classifications. In addition, to reduce errors in the choice of activities that represented a specific intensity, the first and second days of the 7daysRecall WEB assessment included an interactive quiz. The quiz required that the participants match activities with the appropriate intensity, and it demonstrated how the participants were required to complete the assessment. The quiz continued until the participant answered two questions correctly in a row. For scoring, the following METs levels were assigned to each class of activities: sleep = 0.9 METs; light level of activity = 2.2 METs; moderate = 3.5 METs; moderate to high = 4.5 METs; high = 7.0 METs, and very high = 10 METs.

Times of very light-intensity activity, such as reading and television viewing, were not included in the selection screen. Therefore, we subtracted sleep and other activity times from 24 hours and classed activity intensity for this time as very light (= 1.3 METs).

Activity-intensity data for both systems were submitted over the Internet and converted to energy expenditure, which was then transferred to a server. Feedback regarding energy expenditure during the experimental period was not provided to the participants to avoid influencing individual behavior.

### DLW Method

TEE was measured using the previously described DLW method over a 14-day period [7]. On arrival at the testing facility on day 0, participants gave a urine sample for the measurement of baseline^2^H and^18^O enrichment. Between 8:00 and 9:00 AM, a premixed dose containing approximately 0.12 g/kg predicted total body water of^2^H_2_O (99.8 atom%; Taiyo Nippon Sanso, Tokyo, Japan) and 2.5 g/kg predicted total body water of H_2_^18^O (10.0 atom%; Taiyo Nippon Sanso) was given to each participant to drink. Urine samples were collected at the following time points: twice on the next morning (day 1) and twice on the mornings of days 8 and 15. Aliquots of the urine samples were stored frozen at –15°C for later analysis by isotope ratio mass spectrometry (Hydra 20-20 Stable Isotope Mass Spectrometers; SerCon Ltd, Crewe, UK). The gas for equilibration of^18^O was CO_2_, and H_2 _was used for the^2^H. We used platinum catalyst for equilibration of^2^H. Each sample and the corresponding reference were analyzed in duplicate. The average standard deviations for the analyses were 0.7‰ for^2^H and 0.05‰ for^18^O [7].

The^18^O and^2^H dilution spaces (N_O _and N_d_) were determined by dividing the dose of the administered tracer (as moles of^2^H- or^18^O-labeled water) by the intercept method (^2^H and^18^O enrichments at time 0) [20,21]. N_d_/N_O _in the present study was mean 1.027 (SD 0.007), range 1.011-1.043, which is similar to values reported in previous studies [7,22]. Therefore, total body water (mol) was calculated as the mean of N_d _(mol) divided by 1.041 for the dilution space (estimated by^2^H and N_O _[mol]) divided by 1.007 for dilution space (estimated by^18^O) [22].

CO_2 _production rates were determined using the following equation: 0.4554 × total body water × (1.007 ×^18^O elimination rate – 1.041 ×^2^H elimination rate). For this calculation, we used assumptions of isotope fractionation applied to breath water using equation A6 of Schoeller et al [8] with the revised dilution space constant of Racette et al [22]. The TEE calculation was performed using the modified Weir formula [23] based on CO_2 _production rates, and the assumed respiratory quotient was 0.85. PAL was calculated as TEE/BMR [9].

### Measurement of BMR

We measured BMR between 6:00 and 6:30 AM on day 0 of the study using indirect calorimetry (ARCO 2000; ARCO System, Chiba, Japan). CO_2 _production and O_2 _consumption were measured after an overnight fast and 30 minutes of rest. Participants were required to be transported to the laboratory by car to keep physical activity to a minimum. CO_2 _production and O_2 _consumption were converted to BMR through the Weir equation [23]. We used a variation in O_2 _consumption of less than 25 mL/min to determine whether the collection was acceptable [24]. Each participant was monitored periodically to ensure that he or she remained awake. Data were collected in a thermally regulated environment with minimal light and noise. The calorimeter system was calibrated before each measurement.

### Statistical Analysis

All statistical analyses were performed using SPSS for Windows 19.0 (IBM Corporation, Somers, NY, USA). All data are shown as mean (SD); for participant characteristics, a range is also given. We compared men versus women for general characteristics by the independent *t *test.

We calculated zero-order and partial Pearson correlation coefficients as measures of association between AEE measured by DLW and by 24hPAR WEB; TEE measured by DLW and by 24hPAR WEB; PAL measured by DLW and by 24hPAR WEB; AEE measured by DLW and by 7daysRecall WEB; TEE measured by DLW and by7daysRecall WEB; and PAL measured by DLW and by 7daysRecall WEB. Using Bland-Altman plots, we related the difference in AEE between 24hPAR WEB and DLW (y-axis) to the arithmetic mean of AEE for 24hPAR WEB and DLW (x-axis) [25]. In addition, we related the difference in AEE between 7days WEB and DLW (y-axis) to the arithmetic mean of AEE for 7days WEB and DLW (x-axis) [25]. The limits of agreement are given as ±1.96 SD of the difference. To determine the source of error for TEE between 24hPAR WEB and DLW, and for TEE between 7daysRecall WEB and DLW, we calculated the Pearson correlation coefficient to determine the association of the difference between 24hPAR WEB and DLW, and the difference between 7daysRecall WEB and DLW, for TEE, plotted by gender. We defined a significant difference as *P *<. 01.

## Results

Table 1 displays the general characteristics of the study population. We intended to recruit a heterogeneous group (both genders and a wide age and body mass index range), and this is demonstrated in the table. Of the 20 participants, 7 (35%) were overweight (body mass index >25 kg/m^2^). A total of 10 (50%) were employed full-time, 2 (10%) were employed part-time, 4 (20%) were self-employed, and 4 (20%) were full-time homemakers. Men had significantly greater BMRs (*P *< .001), DLW-derived AEE (*P *< .001), and DLW-derived TEE (*P *< .001) than women. There was no significant difference between men and women in PAL, as determined by the DLW method (*P *= .09).

**Table 1:** General characteristics of the study population.

	All participants		Male (n = 10)			Female (n = 10)			*P *value	
	Mean	SD	Mean	SD	Range	Mean	SD	Range	
Age (years)	42.6	10.9	45.2	12.3	26–61	40.0	9.1	25–51	.30
BMI^a ^(kg/m^2^)	24.0	3.8	26.4	3.4	19.2–33.9	21.5	2.4	19.2–27.4	.001
Weight (kg)	66.6	16.3	79.2	13.9	57.5–108.5	54.0	3.6	48.7–58.0	<.001
Height (cm)	165.8	10.4	172.6	7.3	161.9–185.4	159.0	8.5	141.8–171.0	.001
Measured BMR^b ^(MJ)	5.68		6.50	0.89	4.77–8.00	4.87	0.57	4.40–6.24	<.001
TEE_DLW_^c ^(MJ/day)^d^	10.46	2.58	12.39	2.09	8.46–15.50	8.53	1.18	7.41–11.26	<.001
AEE_DLW_^e ^(MJ/day)^d^	3.73	1.35	4.65	1.07	2.85–6.16	2.82	0.89	1.48–4.17	<.001
PAL_DLW_^f ^(TEE/measured BMR)^d^	1.83	0.18	1.90	0.12	1.68–2.09	1.76	0.22	1.42–2.14	.09

^a ^Body mass index.

^b ^Basal metabolic rate.

^c ^ Total energy expenditure measured by doubly labeled water.

^d ^Average value of the total over 2 weeks.

^e ^Activity energy expenditure measured by doubly labeled water.

^f ^Physical activity level measured by doubly labeled water.

**Figure 2:**
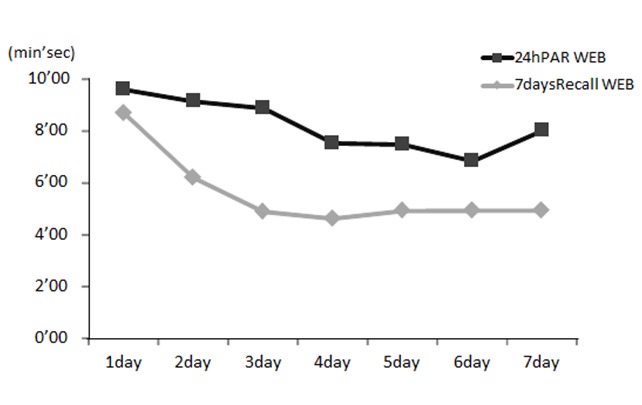
Input time trends (in minutes and seconds for days 1 to 7) for each Web-delivered system. 7daysRecall WEB = 7 days Recall Web, 24hPAR WEB = 24-hour Physical Activity Record Web.

Average input times using the terminal devices were 8 minutes and 10 seconds for the 24hPAR WEB and 6 minutes and 38 seconds for the 7daysRecall WEB system. The average number of input data in the 24hPAR WEB was 8.6 (SD 2.6) types; this refers to the average number of types of behavior, such as reading a book, watching television, and walking. The average number of inputted hours in 7daysRecall WEB was 6.0 (SD 1.5) times; this refers to the average number of inputs for each intensity of activity in the morning, afternoon, and evening. Compliance relating to data input using the terminal devices was 122 of 140 days (87.1%) with the 24hPAR WEB and 133 of 140 days (95.0%) with the 7daysRecall WEB. When the system administrator identified a participant who was not compliant, this was recorded, and the participant received a phone call the next day to remind him or her to complete the input. Using this method, 100% data were collected in this study. Figure 2 shows the response time over the 7 days for each measurement system based on the weblog data. The figure shows that the response time became gradually faster.

The mean AEE was 3.90 (SD 1.43) MJ measured by the 24hPAR WEB method and 3.67 (SD 1.48) MJ by the DLW method. The mean TEE was 10.65 (SD 2.57) MJ by the 24hPAR WEB method and 10.39 (SD 2.68) MJ by the DLW method. Mean PAL was 1.87 (SD 0.20) by 24hPAR WEB and 1.82 (SD 0.22) by DLW. Mean AEE was 4.29 (SD 1.94) MJ by the 7daysRecall WEB method and 3.80 MJ (SD 1.36) by DLW. The mean TEE was 11.08 (SD 2.82) MJ by the 7daysRecall WEB method and 10.53 (SD 2.58) MJ by DLW, and mean PAL was 1.96 (SD 0.32) for the 7daysRecall WEB method and 1.84 (SD 0.19) by DLW. There were no significant differences between the Web-based measurements and the DLW method for the above energy expenditure variables.

**Figure 3:**
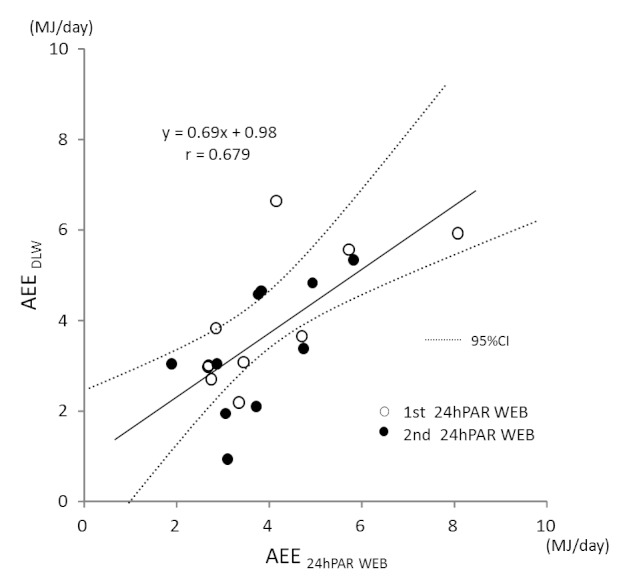
Pearson correlation coefficient for daily activity energy expenditure (AEE) measured by the 24-hour Physical Activity Record WEB (24hPAR WEB) and doubly labeled water (DLW) methods. CI = confidence interval.

**Figure 4:**
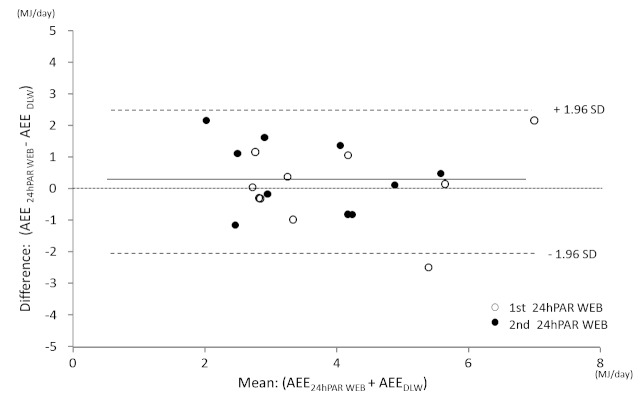
Bland–Altman plot illustrating the difference in activity energy expenditure (AEE) between the 24-hour Physical Activity Record Web (24hPAR WEB) and the doubly labeled water (DLW) methods.

Figure 3 shows the Pearson correlation coefficient between the 24hPAR WEB and DLW methods for daily AEE. AEE measured by 24hPAR WEB and by DLW was correlated (*r *= .679, *P *< .001). Correlation coefficients for TEE and PAL by the 24hPAR WEB and the DLW methods were *r *= .874 (*P *< .001) and *r *= .404 (*P *= .08), respectively. Figure 4 shows the Bland-Altman plot for AEE as measured by 24hPAR WEB compared with DLW. The mean difference for the 24hPAR WEB and DLW methods was small (0.23 MJ), and the limits of agreement were 2.33 MJ (±1.96 SD). The test for trend was not statistically significant. The regression equation was y = –0.03x + 0.36 (*r *= –.382, *P *= .87). In the Bland-Altman plot for TEE measured by 24hPAR WEB compared with DLW, the mean difference between the two methods was small (0.26 MJ), and the limits of agreement were 2.59 MJ (±1.96 SD). The test for trend was not statistically significant. The regression equation was y = –0.04x + 0.72 (*r *= –.085, *P *= .72).

The Pearson correlation for AEE between the 7daysRecall WEB and DLW methods was *r *= .144 (*P *= .54). TEE as measured by 7daysRecall WEB and DLW was correlated (*r *= .590, *P *= .006). The correlation coefficient for PAL as measured by the 7daysRecall WEB and DLW methods was *r *= –.085, with no significant correlation.

**Figure 5:**
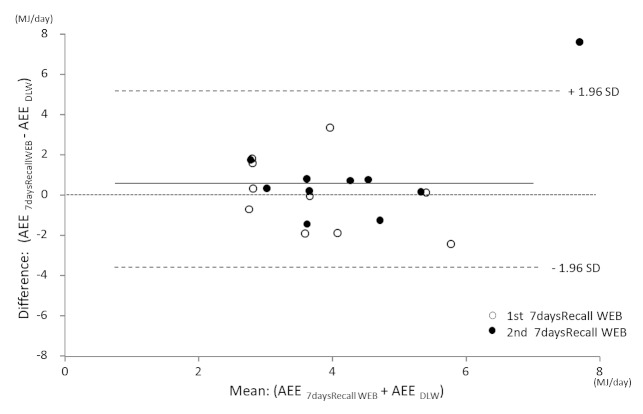
Bland–Altman plot illustrating the difference in activity energy expenditure (AEE) between the 7 days Recall Web (7daysRecall WEB) and the doubly labeled water (DLW) methods.

Figure 5 shows the Bland-Altman plot for AEE as measured by 7daysRecall WEB compared with DLW. The mean difference for the 7daysRecall WEB and the DLW method was small (0.49 MJ), and the limits of agreement were large at 4.32 MJ (±1.96 SD). The test for trend was not statistically significant. The regression equation was y = 0.59x – 1.93 (*r *= .343, *P *= .14). In the Bland-Altman plot for TEE measured by 7daysRecall WEB compared with DLW, the mean difference between the two methods was small (0.55 MJ), and the limits of agreement were large at 4.80 MJ (±1.96 SD). The test for trend was not statistically significant. The regression equation was y = 0.11x – 0.66 (*r *= .110, *P *= .65).

**Figure 6:**
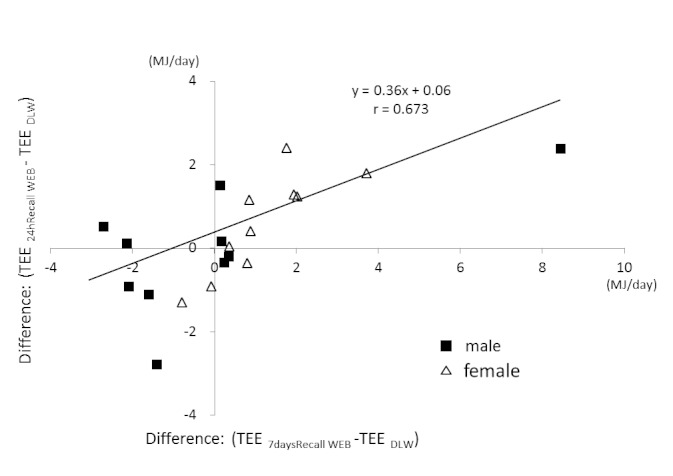
Correlation of the difference between the doubly labeled water (DLW) and 24-hour Physical Activity Record Web (24hPAR WEB) measures of total energy expenditure (TEE) and the difference between the DLW and 7 days Recall Web (7daysRecall WEB) measures of TEE.

Figure 6 shows the correlation of the difference between the DLW and 24hPAR WEB measures of TEE and the difference between the DLW and 7daysRecall WEB measures of TEE (*r *= .673, *P *= .001). The figure demonstrates the profile of the methods regarding overestimation or underestimation of TEE. Most of the women and 1 man overestimated TEE in both the 24hPAR WEB and the 7daysRecall WEB methods (Figure 6).

## Discussion

In this study, we developed a new physical activity assessment method using Web-based measurement systems and validated them against the DLW method. These measurement systems are low in cost, practical, and user friendly. They can be completed quickly, with an input time of less than 10 minutes. With both systems, the daily response time tended to become progressively faster; this was particularly the case with the 7daysRecall WEB method. It is possible that increased operational familiarity aided quicker recall of behavior. The systems can measure daily physical activity in many people simultaneously because they are compatible with many terminal devices, such as cell phones, smartphones, and personal computers, allowing complete assessments to be made via the Internet. These two systems were an improvement compared with the diary methods [14] and interview methods [15] developed previously.

Diary methods for the assessment of physical activity have been used since the 1960s [26]. The method requires that individuals record their physical activity behavior every 15 minutes. We used this method in a previous study [27], though the activities selected for the present study were modified from a previous study [14]. An advantage of the Web-based measurement systems used in the present study is that they are compatible with a variety of terminal devices. They also allowed for a greater variety of physical activity behaviors to be assessed, and they simplified the recording of data.

In the present study, we found a significant association between 24hPAR WEB and DLW measurements of AEE. This result is important because BMR accounts for 60%-75% of TEE [6]. In addition, the relationship between 24hPAR WEB and DLW measurements of TEE was similar to that between 3-axis accelerometry and DLW measurements of TEE [7]. This suggests that TEE can be assessed accurately in under 10 minutes using the 24hPAR WEB. The 24hPAR WEB allows for the assessment of swimming, cycling, and climbing activities, which cannot be measured using an accelerometer. The 24hPAR WEB can also classify such activities as traffic behavior, sleeping, working, and leisure time.

The correlation between 24hPAR WEB and DLW measurements of TEE in the present study was similar to that found in previous research [14]. The present study also demonstrated that physical activity can be reported on a single digital screen without the need for cross-tabulation from several pieces of paper. A significant finding was that the accuracy of 24hPAR WEB measurement of AEE was superior to that in conventional physical activity questionnaires when compared with the DLW method. Bonn et al reported a Spearman correlation between Active-Q, a Web-based questionnaire, and DLW measurements of TEE of *r *= .52 (*P *<.001, n = 37) [11]; similarly, Ishikawa-Takata et al reported a Spearman correlation between Japan Arteriosclerosis Longitudinal Study Physical Activity Questionnaire and DLW measurements of TEE of *r *= .742 (*P *<.001, n = 226) [28]. A weakness of the present study was that we had fewer participants than in previous studies; however, we saw a moderately high correlation even with AEE (Figure 3). Our study findings confirm that the diary method is the most accurate for self-reporting.

Figure 4 indicates that the error range of the estimate did not change regardless of the highest and lowest average values for AEE. Because there was a meaningful correlation with the DLW methods, and the error range was small (±2.33 MJ, 95% confidence interval) regardless of the size of the estimate, we found that the 24hPAR WEB method provided better results than methods used in previous studies [11,14]. When using assessment tools that require greater effort for recalling behavior, such as the Active-Q [11] and the 7daysRecall WEB in the present study, study participants have been found to over- or underestimate their physical activity. With regard to the time of each behavior, they may be able to recall impressive behavior but unable to recall nonimpressive behavior. However, for the 24hPAR WEB, we observed no significant over- or underestimation. One reason for this finding may be that selecting the order of behavior after awaking results in greater accuracy. Another reason may be that behaviors were selected every 15 minutes.

The 7-day physical activity recall method was originally developed in 1979 for use in the Stanford Five-City Project [15]. An advantage of this method is that the previous 7 days of physical activity could be estimated from a 15-minute interview [15]. For the present study, we were unable to develop an interactive communication system that could simulate an interview. Therefore, we devised a method of effective communication in a manner that obtained similar information to that obtained in a face-to-face interview. This method included the following: (1) input once per day recalling that day’s activities, compared with the original 7-day physical activity recall frequency of once per week, (2) illustrations of physical activity intensity, and (3) quizzes over the first 2 days that required participants to select the intensity among activities (Multimedia Appendix 2). The quiz questions continued until the participants were able to answer two questions correctly in a row. Those who did not look at the illustrations were unable to answer correctly; consequently not allowing them to continue until they answered correctly reduced potential mistakes in the self-reported exercise intensity of their activities.

Compared with previous studies that examined the 7-day recall [29-31] method, the 7daysRecall WEB predicted TEE accurately. Figure 5 indicates that the error range of the estimate would not change regardless of the high and low average value of AEE. The 95% confidence interval was ± 4.32 MJ, demonstrating a large variation compared with 24hPAR WEB. However, the error was small compared with the 7-day recall diary method [29-31], and it was similar to that of the Active-Q [11].

In using the 7daysRecall WEB, 1 man greatly overestimated (3SD above the average) energy expenditure. Therefore, we excluded his data and reanalyzed the data (n = 19). The recalculated Pearson correlation coefficient for TEE between the 7daysRecall WEB and DLW methods was *r *= .788 (*P *<.001). AEE measured by 7daysRecall WEB and DLW was not significantly correlated (*r *= .346); nor was and PAL measured by 7daysRecall WEB and DLW (*r *= -.001). After completion of the study, we found that the reason for energy expenditure being overestimated by the male participant was that, regardless of his physical activity, the intensity level he inputted corresponded to light-intensity child care time. To prevent this type of mistake, we programmed the system to request that the participant confirm each input. However, accurate recording of physical activity behavior appears to be a limitation of self-reported physical activity assessment methods [32].

The present study employed a crossover design, whereby participants alternately used two Web-based methods. With respect to response time, there was no difference (*P *= .32) between the group that used 24hPAR WEB during the first week (450, SD 163 seconds) and the group that used 24hPAR WEB during the second week (596, SD 329 seconds). There was likewise no difference (*P *= .68) between the group that used 7daysRecall WEB during the first week (353, SD 225 seconds) and the group that used 7daysRecall WEB during the second week (318, SD 140 seconds). In Figure 3, Figure 4, and Figure 5, correlation charts present the validity of the Web-based methods compared with the DLW method. No difference in the validity was evident as a result of using the crossover design. However, future investigations should examine this issue of the crossover design, since we had just 10 participants in each group.

With both systems, women tended to overestimate energy expenditure (Figure 6). Of the 7 women who made such overestimations, 6 were homemakers or were employed part-time. A possible reason for this estimation relates to the difficulty in determining the intensity of child care and housework activity. Housework and child care require complex physical activity. It is possible that, rather than selecting the time spent moving, the participants selected the time engaged in housework and child care. However, further study is required to investigate this issue.

Another limitation of this study was that conventional cell phones cannot support JavaScript. Therefore, conventional cell phones could not operate the 24hPAR WEB. In Japan, 95% of people aged 30-49 years have access to the Internet, whereas just 32.9% of those aged 70-79 years have such access [33]. Therefore, sampling is an important issue when using the 24hPAR WEB for epidemiological studies in the elderly. Compliance during the 7-day input is also an important consideration for obtaining accurate measurements.

In recent years, public health research has focused on disease prevention and community intervention to promote physical activity [34]. Over the last decade, many programs have also developed Web-based interventions to help increase physical activity [35,36]. For research targeting large groups of individuals, evaluation of physical activity is important in determining the effects of interventions. The Web-based measurements developed in the present study will be useful for accurately assessing physical activity at low cost.

In conclusion, the 24hPAR WEB appears to be valid for estimating AEE and TEE, and the 7daysRecall WEB appears to be valid for estimating TEE. Both methods are effective for collecting physical activity data in epidemiological studies. The 24hPAR WEB is more accurate than the 7daysRecall WEB, and it is useful for evaluating physical activity behavior and the effect of interventions. The input time for the 7daysRecall WEB is short. The system is easy to operate and suitable for evaluating physical activity in large communities.

## Multimedia Appendix 1

24h Physical Activity Record WEB.

## Multimedia Appendix 2

7daysRecall WEB.

## Abbreviations

7daysRecall WEB: 7 days Recall Web
24hPAR WEB: 24-hour Physical Activity Record Web
AEE: activity energy expenditure
BMR: basal metabolic rate
DLW: doubly labeled water
METs: metabolic equivalents
Nd: 2H dilution space
NO: 18O dilution space
PAL: physical activity level
TEE: total energy expenditure
