# Supplementary material for: Validation of Web-Based Physical Activity Measurement Systems Using Doubly Labeled Water
Source: J Med Internet Res. 2012 Sep 25;14(5):e123. doi: 10.2196/jmir.2253 (PMC3517333; doi:10.2196/jmir.2253)
Supplement: Supplementary file 1 [file jmir_v14i5e123_app1.pdf]

Appendix I□24h Physical Activity Record WEB

Looking back over the past 24 hours, for each 15 minute period, select and record the activities in the table on the right of the page.

Sample screen

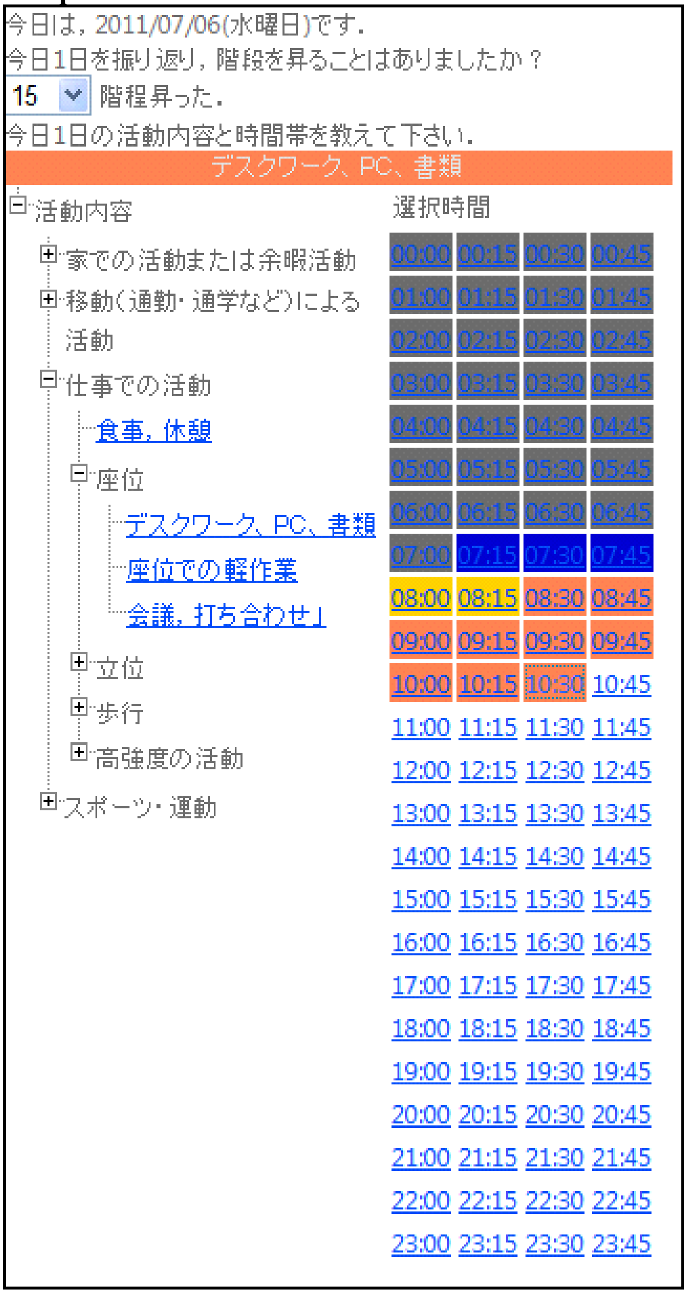

Appendix I
Items included in the 24h physical Activity Record WEB and their metabolic equivalents

Type of behavior	METs
**Sleeping**	0.9
**Leisure time activities**	
Eating, reading, newspaper, TV	1.3
Brushing teeth, makeup, bath	2.1
Walk (Paseo)	2.1
Cleaning, cooking, washing	2.3
Foods shopping, childcare, walking	2.8
Window cleaner, cutting grass	4.1
Car wash (wax), DIY	4.5
Hole digging, shoveling snow	5.5
**Way to work**	
Walking slowly	2.5
Normal walking	3.5
Brisk walking	4
About 15km/h bicycle slowly	4
About 16-19km/h bicycle natural	6
About 19-23km/h bicycle fast	10
About 23-26km/h bicycle very fast	12
Train (sitting position)	1.3
Train (standing)	2
Bus (sitting position)	1.3
Bus (standing)	2
Car	1.3
Motorbike	1.5
**Work related activities**	
Meals, break	1.3
Desk work, PC, documentation	1.5
Light work in a sitting position	2.5
Light work, in-store customer service	2.5
Work of moderate intensity, shelf arrangement	3
High-intensity work, cargo	4
Walking slowly	2.5
Brisk walking	4
Lightweight movement of goods (such as a dish)	4
Weight movement of goods (eg furniture)	5
Painting	4.5
Digging	5.5
Agricultural, construction industry	6
Heavy cargo	7
Conference, meeting	1.5

Appendix I Continued

Type of behavior	METs
**Sports activities**	
Walking slowly	2.5
Normal walking	3.5
Brisk walking	4
Jogging slowly	7
Mountain climbing, hiking	7.5
Jogging Slightly faster	8
Running fast	12
About 15km/h bicycle slowly	4
About 16-19km/h bicycle natural	6
About 19-23km/h bicycle fast	10
About 23-26km/h bicycle very fast	12
Fishing (sitting position)	2.5
Fishing (standing)	3.5
Fishing (mountain stream)	6
Golf (practice field)	3
Golf (with cart)	3.5
Golf (no cart)	4.5
Play catch	2.5
Bowling	3
Badminton	4.5
Baseball, softball	5
Soccer, tennis	7
Stretch	2.5
Strength training (light and moderate intensity)	3
Jump rope	5
Strength training (high intensity)	6
Skiing	7
Swimming (slowly)	8
Swimming (faster)	12
