# Supplementary material for: Validation of Web-Based Physical Activity Measurement Systems Using Doubly Labeled Water
Source: J Med Internet Res. 2012 Sep 25;14(5):e123. doi: 10.2196/jmir.2253 (PMC3517333; doi:10.2196/jmir.2253)
Supplement: Supplementary file 2 [file jmir_v14i5e123_app2.pdf]

Appendix II□7daysRecall WEB

Instructions
In this study, the answers are divided into three sections over one day.
Definition of the morning from wake up to lunch
Definition of the afternoon from lunch to dinner
Definition of the night from the dinner to bed time

Recall activities in each category. Please select a different time for exercise intensity.
Exercise intensity is a choice of five "light" "moderate" " moderate /high" " high" " very hard".
Definitions of intensity and activities, please always check the Appendix table with illustrations.
If you have activities that are not listed in the table, choose similar activities and activities that are listed in the table and please select the exercise intensity.

Interactive intensity quizzes (the first and second day)
For the answer look at the table in the Appendix that has illustrations

Q. 1 "Walking" Which of the following applies to the intensity?

- light
- moderate
- moderate /high
- high
- very hard

Q [illegible] "Cleaning, washing, cooking" Which of the following applies to the intensity?

- light
- moderate
- moderate /high
- high
- very hard

□[illegible]e above two questions are an example. Keep answering the questions until you answer two questions correctly in a row.

Appendix II

Today is Monday the 1st of September.

We would like to ask you about your sleep time. What time did you go to bed last night? What time did you wake up this morning?

Bedtime [ ] h [ ] min
Wake-up time [ ] h [ ] min

Where did you go today? What did you do? Did you do anything special? Please try and recall your activities as accurately as possible. Please select the time that you actually spent moving your body whilst performing the following activities.

The selections below refer to the activities you performed in the morning (from wake up to lunch).

light [ ] hours [ ] min

[ ] hours [ ] min

[ ] hours [ ] min

moderate

moderate /high

high

very hard

Confirm

The selections below refer to the activities you performed in the afternoon (from lunch to dinner).

light hours min

moderate hours min

moderate /high hours min

high hours min

very hard hours min

Confirm

Appendix II

hours min

The selections below re[illegible] activ[illegible] u performed in the night (from the dinner to bed time).

light hours min

moderate hours min

moderate /high hours min

high hours min

very hard hours min

Confirm

Look back over yesterd[illegible] activity, d[illegible] forget to include any activity?
Did you move your bod[illegible] hours [illegible] min ng recreational sports or activity such as walking?

- You want to modify the activities of the morning
- You want to modify the activities of the afternoon
- You want to modify the activities of the night
- The activities are all correct

Confirm

Today's input is completed.
We ask for your cooperation in the remaining six days. Thank you.

Appendix II

Table indicating physical activity intensity illustrations

Category	METs	Activities	Illustration of image
sleep	0.9	sleeping	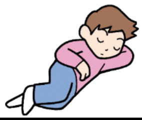
light	2.2	Home (cleaning, washing, cooking, shopping, walking, brushing teeth, shower, etc.)	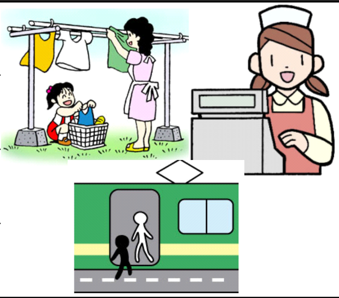
		Work (in-store sales activities,light work in a standing position, etc.)	
		Transport □train and bus ride in a standing position□	
		Sports exercise (stretching, light exercise)	
moderate	3.5	Home (window cleaner, cutting grass, gardening, etc.)	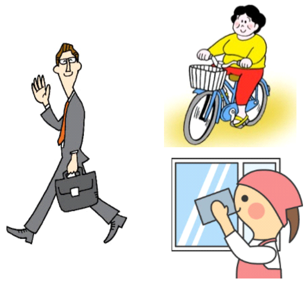
		Work (shelf arrangement, cargo, brisk walking, movement of dishes and tray, etc.)	
		Transport □Walking, about 15km/h bicycle slowly□	
		Sports exercise (walking, golf, etc.)	
moderate /high	5	Home □DIY, digging, shoveling snow, etc.□	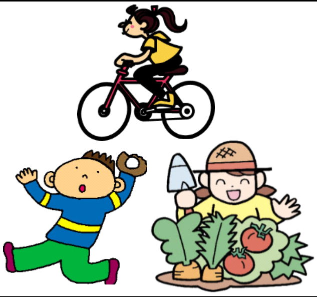
		Work (digging, agricultural, etc.)	
		Transport□about 16-19km/h bicycle natural□	
		Sports exercise□baseball, softball, strength training, etc.□	
high	7	Work□Dig a ditch with a shovel, truck cargo luggage, etc.)	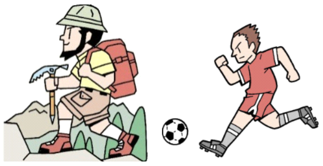
		Transport□about 19-23km/h bicycle fast□	
		Sports exercise□7-8km/h jogging slowly, soccer, tennis, mountain climbing, etc.)	
very hard	10	Transport□about 23-26km/h bicycle very fast□	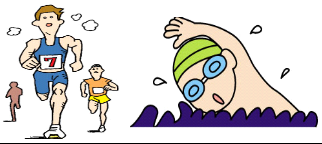
		Sports exercise□7-8km/h jogging fast, swimming, etc.)	
